# Supplementary material for: Clinical Potential of Circulating Cell-Free DNA (cfDNA) for Longitudinally Monitoring Clinical Outcomes in the First-Line Setting of Non-Small-Cell Lung Cancer (NSCLC): A Real-World Prospective Study
Source: Cancers (Basel). 2022 Dec 6;14(23):6013. doi: 10.3390/cancers14236013 (PMC9735435; doi:10.3390/cancers14236013)
Supplement: Supplementary file 1 [file cancers-14-06013-s001.zip › cancers-2008211-supplementary.pdf]

cancers

Supplementary Material: Clinical Potential of Circulating Cell-Free DNA (cfDNA) for Longitudinally Monitoring Clinical Outcomes in the First-Line Setting of Non-Small-Cell Lung Cancer (NSCLC): A Real-World Prospective Study

Valerio Gristina, Nadia Barraco, Maria La Mantia, Luisa Castellana, Lavinia Insalaco, Marco Bono, Alessandro Perez, Delia Sardo, Sara Inguglia, Federica Iacono, Sofia Cutaia, Tancredi Didier Bazan Russo, Edoardo Francini, Lorena Incorvaia, Giuseppe Badalamenti, Antonio Russo, Antonio Galvano, Viviana Bazan

Supplementary Material Methods. FFPE nucleic acids extraction and molecular analysis.

The mutational status for the detection of oncogene addiction was tested on the thickness of 10 µm tissue sections obtained from biopsy at baseline or resected tumor tissue stored as FFPE samples. PD-L1 immunohistochemistry (IHC) was carried out on 4-µm sections of FFPE tumor tissue samples using Dako PD-L1 IHC 28-8 PharmaDx (Agilent) and evaluated by a trained pathologist according to the tumor proportion score (TPS), defined as the percentage of positive viable tumor cells among all viable tumor cells evaluated.

DNA and RNA nucleic acids were extracted from six 10 µm thickness FFPE tissue sections with an adequate percentage of neoplastic cells ≥20%. The genomic DNA and RNA were extracted from FFPE tissue using the QIAmp FFPE Tissue Kit and RNeasy FFPE kit (Qiagen, Hilden, Germany), respectively and quantified in terms of ng/µl using Qubit™ dsDNA HS Assay Kit and Qubit™ RNA HS Assay Kit (ThermoFisher Scientific, Foster City, CA, USA), respectively. According to clinical practice, 10 nanograms of both DNA and RNA including *EGFR*, *BRAF*, *KRAS*, *ALK*, *ROS1* genes alterations, *MET* amplification, and eventually the gene fusion transcripts were tested using the Oncomine™ Focus DNA/RNA panel. Moreover, the Oncomine™ RNA assay offered the opportunity to evaluate the 5'/3'imbalance ratio at the *ALK*, *ROS1*, *RET* and *NTRK1* genes as a fusion signature independently by the unknown partner. Libraries were quantified by Ion Library TaqMan™ quantification kit on QuantStudio7 Pro Real-Time PCR System (Applied Biosystem) using Design&Analysis Software v2.4.3. The analytical sensitivity of the assay achieved at an allelic frequency ≥5% was 100%, but the performance of every single run was referred to the data. The data were tested on an amplicon-based sequencing platform Ion Torrent S5™ System. The Oncomine Focus-520-w.30-DNA-Single Sample and the Oncomine Focus-520-w.30-Fusions-Single Sample represented the workflow applied for the analysis of DNA and RNA samples. To test the reliability of data for DNA sequencing, we complied with the following thresholds: mapped read >300,000.00, mean read length >75 bp, uniformity >90%, and mean raw accuracy >99%. For RNA sequencing, we considered acceptable an analysis with mapped read >50.000, mean read length >60 bp, and expression controls detected ≥3 out of 5. All reagents, kits and platforms used were provided by ThermoFisher Scientific, Foster City, CA, USA. The data of DNA sequencing were analyzed with Ion Torrent TorrentSuite™ (TS, version 5.18) processing the plug-in of Coverage Analysis and Variant Caller. Integrative Genomics Viewer (IGV v2.4.1) was used to visibly evaluate the alignments of sequences. Pathogenetic changes in DNA, and RNA sequences with the related percentage of allelic frequency were annotated, only for DNA analysis, by Ion Reporter Software v5.18 applying the filter chains Oncomine variants for default use and DefaultFusion View 5.18, respectively, and were described using the Human Genome Variation Society (HGVS) standard nomenclature. To confirm the data of common pathogenic variants or the cases of poor

quality and quantity DNA, 15-30 ng of DNA was amplified using EasyPGX ready EGFR/BRAF/KRAS kit with a LOD of 5%. After about 2 hours run, the data obtained on Real time EasyPGX System (Diatech Pharmacogenetic, Jesi AN-Italy) were analyzed using EasyPGX Analysis Software v4.0.10.

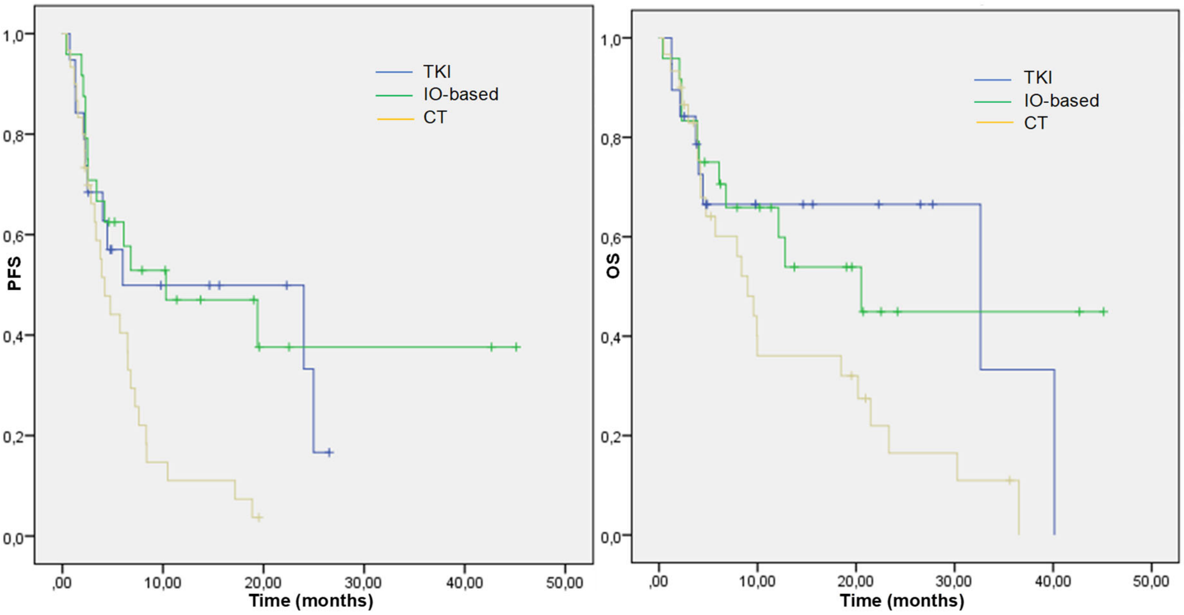

Figure S1. Kaplan–Meier analysis of PFS and OS in NSCLC patients according to treatment subgroups. TKI, tyrosine kinase inhibitor; IO, immune-oncology; CT, platinum-based chemotherapy; NSCLC, non-small cell lung cancer; PFS, progression-free survival; OS, overall survival

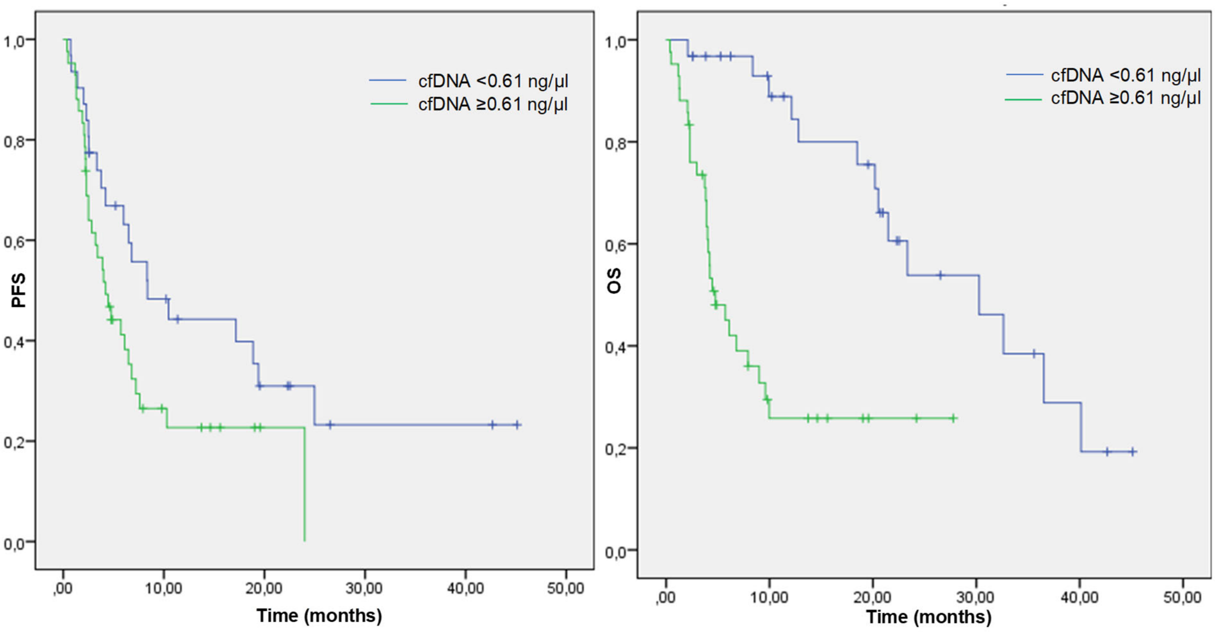

Figure S2. Kaplan–Meier analysis of PFS and OS according to the median cfDNA value in the overall cohort population. cfDNA, cell-free DNA; PFS, progression-free survival; OS, overall survival.

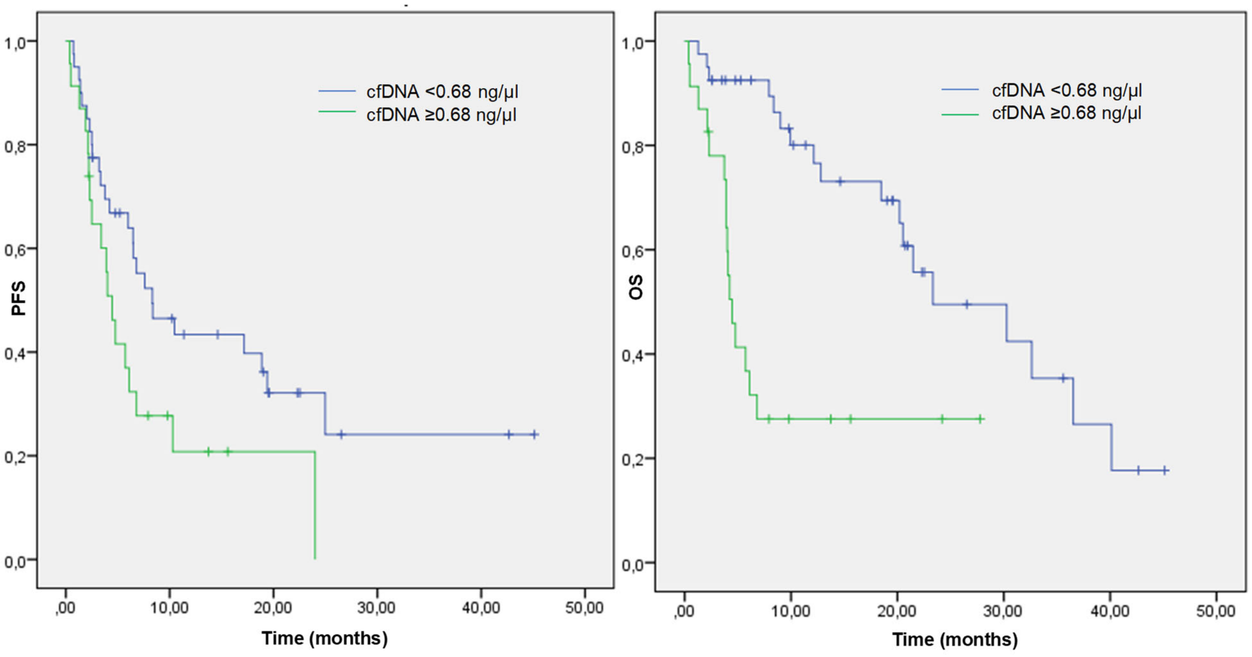

Figure S3. Kaplan–Meier analysis of PFS and OS according to the cfDNA cut-off based on X-tile analysis in the overall cohort population. cfDNA, cell-free DNA; PFS, progression-free survival; OS, overall survival.

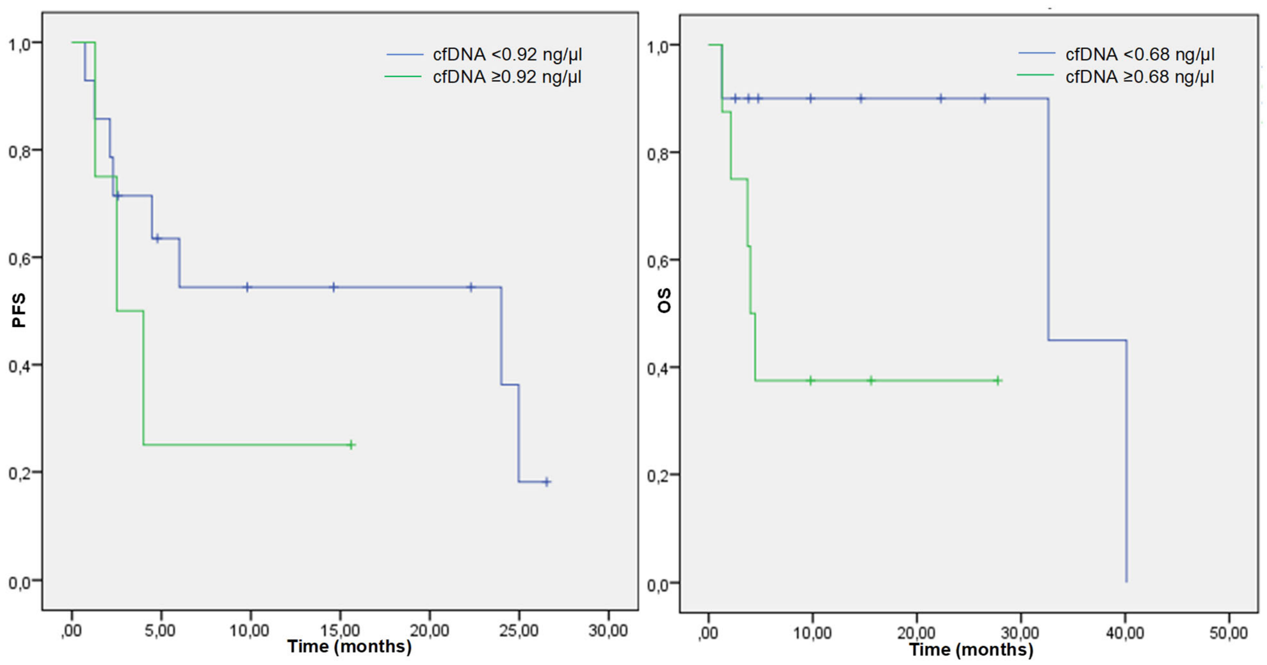

Figure S4. Kaplan–Meier analysis of PFS and OS according to the cfDNA cut-off based on X-tile analysis in patients with NSCLC receiving TKIs. cfDNA, cell-free DNA; PFS, progression-free survival; OS, overall survival; NSCLC, non-small cell lung cancer; TKI, tyorisine kinase inhibitor.

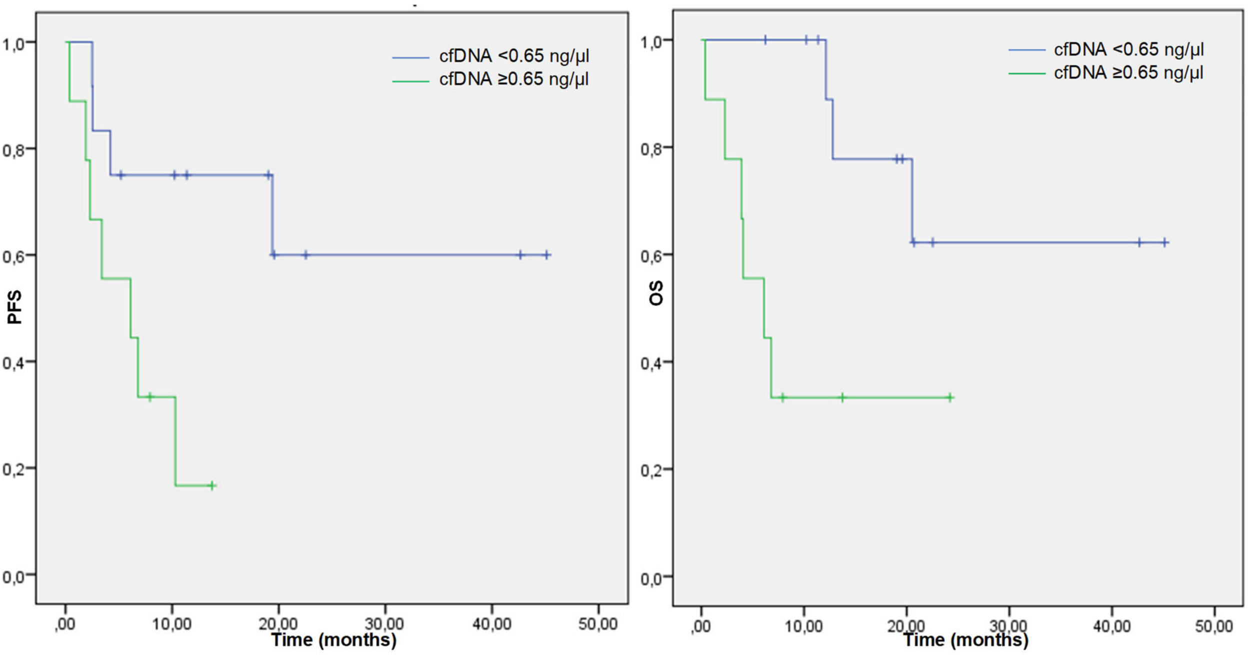

Figure S5. Kaplan–Meier analysis of PFS and OS according to the cfDNA cut-off based on X-tile analysis in patients with NSCLC receiving IO-based treatments. cfDNA, cell-free DNA; PFS, progression-free survival; OS, overall survival; NSCLC, non-small cell lung cancer; IO, immune-oncology.

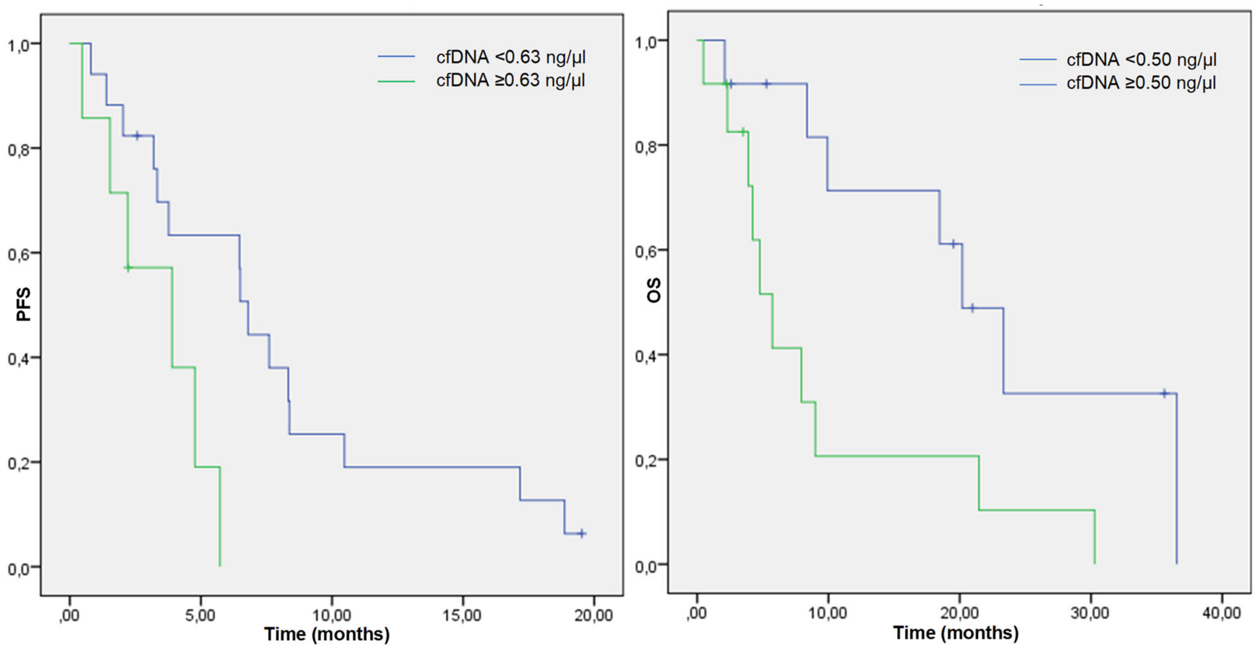

Figure S6. Kaplan–Meier analysis of PFS and OS according to the cfDNA cut-off based on X-tile analysis in patients with NSCLC receiving CT. cfDNA, cell-free DNA; PFS, progression-free survival; OS, overall survival; NSCLC, non-small cell lung cancer; CT, platinum-based chemotherapy.

Table S1. Univariate and multivariate analysis of progression-free survival (PFS).

			Univariate			Multivariate	
	**N**	**Events**	**PFS (median months)**	**95% CI**	**p (log rank)**	**p**	**HR (95% CI)**
**Sex**					0.088		
M	52	41	4.8	2,77-6,76			
F	21	10	17.2	1,99-32,34			
**Age**					0.282		
<65	33	22	6.8	1,15-12,07			
≥65	40	29	5.7	3,23-8,22			
**ECOG PS**					**0.029**		
0	27	16	10.3	0-22,78			
1	13	10	5.7	1,85-9,60			
2	33	25	4.2	2,33-6,06			
**Smoking status**					0.094		
Never	13	7	17.2	2,25-32,09			
Current/former	56	40	6.1	3,95-8,25			
**Histology**					0.517		
non-squamous	60	41	6.0	3,72-8,27			
squamous	13	10	6.5	2,26-10,67			
**Brain metastasis**					0.957		
No	59	41	6.0	3,11-8,89			
Yes	14	10	6.1	2,57-9,63			
**Bone metastasis**							
No	46	30	6.8	4,02-9,58			
Yes	27	21	5.7	2,7-8,76			
**Liver metastasis**					**<0,0001**	**<0,0001**	0,027 (0,004-0,175)
No	61	41	6.8	4,93-8,67			
Yes	10	9	1.3	0-2,63			
**Adrenal metastasis**					**<0,014**		
No	59	39	6.8	4,27-9,33			
Yes	14	12	2.2	0-5,33			
**Site of disease**					**0.248**		
Intra-thoracic	22	14	8.3	6,28-10,38			
Extra-thoracic	49	36	4.5	2,23-6,71			
**Median cfDNA**					**0.043**		
<0,61	31	20	8.4	2,46-14,27			
≥0,61	42	31	4.2	2,51-5,88			
**cfDNA increase**					**0.004**	**0.005**	0,345 (0,165-0,722)
<20%	27	14	18.9	6,25-31,48			
≥20%	20	17	3.3	2,89-3,76			

Table S2. Univariate and multivariate analysis overall survival (OS).

	N	Events	Univariate			Multivariate	
			OS (median months)	95% CI	p (log rank)	p	HR (95% CI)
**Sex**					0.079		
M	52	34	9.9	5,68-14,25			
F	21	8	40.1	0-85,59			
**Age**					0.51		
<65	33	19	21.5	15,87-27,06			
≥65	40	23	9.9	7,37-12,48			
**ECOG PS**					**<0,0001**		
0	27	11	36.0	28,60-43,39		**0.004**	**0,226 (0,083- 0,614)**
1	13	9	20.2	7,36-33,03		0.597	0,79 (0,330-1,890)
2	33	22	6.1	2,48-9,71			1
**Smoking status**					0.16		
Never	13	6	32.6	0-73,06			
Current/former	56	32	12.8	1,87-23,73			
**Histology**					0.715		
non-squamous	60	35	10.0	0-25,48			
squamous	13	7	18.5	1,9-35,03			
**Brain metastasis**					0.22		
No	46	23	20.5	6,85-34,2			
Yes	27	19	9.0	3,14-14,8			
**Bone metastasis**					0.063		
No	46	23	20.5	6,85-34,2			
Yes	27	19	9.0	3,14-14,85			
**Liver metastasis**					**0.012**	**0.004**	0,314 (0,141-0,697)
No	61	33	18.5	6,95-29,98			
Yes	10	9	2.1	0-4,71			
**Adrenal metasta-sis**					**<0,0001**		
No	59	31	20.5	10,96-30,10			
Yes	14	11	3.9	2,01-5,78			
**Site of disease**					**0.017**		
Intra-thoracic	22	10	30.3	16,45-44,08			
Extra-thoracic	49	32	9.6	3,35-15,84			
**Median cfDNA**					**<0,0001**	**0.001**	0,212 (0,088-0,510)
<0,61	31	14	30.3	18,38-42,15			
≥0,61	42	28	4.8	2,48-7,05			
**cfDNA increase**					0.129		
<20%	27	11	30.3	12,21-48,32			
≥20%	20	13	20.5	14,44-26,62			
